# Thrombocytopenia in Pregnancy: Identification and Management at a Reference Center in Pakistan

**DOI:** 10.7759/cureus.23490

**Published:** 2022-03-25

**Authors:** Munira Borhany, Madiha Abid, Sidra Zafar, Uzma Zaidi, Saima Munzir, Tahir Shamsi

**Affiliations:** 1 Hematology, National Institute of Blood Diseases and Bone Marrow Transplantation, Karachi, PAK; 2 Research and Development, National Institute of Blood Diseases and Bone Marrow Transplantation, Karachi, PAK

**Keywords:** thrombocytopenia, pregnancy, platelet transfusion, immune thrombocytopenic purpura, gestational, eclampsia

## Abstract

Objective: The study aimed to evaluate the causes of thrombocytopenia in pregnancy and its management along with the outcome in the COVID-19 era.

Methods: Recruitment for this prospective, cross-sectional observational study of thrombocytopenia in pregnancy (platelet counts <100x10^9^/L) was done from January 2017 to August 2020 at the National Institute of Blood Diseases (NIBD) after taking the patients’ informed consent. Complete clinical and lab profile of patients was also collected.

Results: A total of 150 pregnant women with thrombocytopenia were enrolled, with the mean age being 27.3±4.64 years. Mean platelet counts at baseline were 48.0±24. Main clinical manifestations at baseline included: anemia 65.9%, bruises 23.25%, and edema 9.3%. Causes of thrombocytopenia were gestational thrombocytopenia (GT) 72 (48%), acute fatty liver five (3.3%), pre-eclampsia in 11 (7.3%), and eclampsia seven (4.6%). Causes not specific to pregnancy included 30 (20%) cases of ITP, hepatitis C, and nutritional deficiency was reported in nine (6%) patients each. 72/150 received supportive care treatment to manage thrombocytopenia and were closely monitored and given supplements. Twenty (66.6%) ITP patients received treatment with steroids, with complete response in 70% of them seen. Overall, 38 (25.3%) women with bleeding symptoms and platelet count <50x10^9^/L received platelet transfusions.

Conclusion: The study shows that pre-eclampsia and eclampsia are serious conditions with a high risk for complications, while GT is a benign and the most common cause of thrombocytopenia in pregnancy which requires no active treatment. The other causes such as ITP and infections require individualized management.

## Introduction

Thrombocytopenia is the second leading cause of blood disorders in pregnancy after anemia and complicates about 7% to 10% of all pregnancies. It is defined as a platelet count of less than 100x10^9^/L and classified as mild (100-50x10^9^/L), moderate (50-30x10^9^/L), and severe (less than 30x 10^9^/L). The explanation for the drop in platelet count during normal pregnancy is unknown, however, it could be due to lower platelet production or increased platelet “turn over” during pregnancy, which is most likely from hemodilution related to an increase in plasma volume during pregnancy and possibly increased platelet clearance as mean platelet volumes, platelet volume distribution width, and platelet-derived cyclooxygenase products rise [1,2].

Pregnancy adds to the urgency of establishing the cause of thrombocytopenia and making extra care options due to the possibility of problems affecting both the patient and the fetus. Furthermore, the causes of thrombocytopenia differ depending on the duration of gestation, the severity of the thrombocytopenia, and the patient's health status.

According to the literature, gestational thrombocytopenia (GT) with platelet counts less than 100x10^9^/L occurs in 4.4% to 11.6% of pregnancies, accounting for almost 75% of all occurrences of thrombocytopenia in pregnancy, pre-eclampsia with severe features/HELLP syndrome 22%, immune thrombocytopenic purpura (ITP) accounted for 11%, whereas other causes accounted for 8% (includes antiphospholipid syndrome, disseminated intravascular coagulation, dilutional thrombocytopenia, myeloproliferative neoplasm, and nutritional deficiencies) [3-5]. The assessment of thrombocytopenia is critical to rule out any systemic illnesses that may affect pregnancy care, as thrombocytopenia can appear as an isolated finding or in conjunction with other conditions. Previous research in Pakistan has shown that the most common cause of thrombocytopenia in pregnancy is GT, which affects around 6% of pregnancies and accounts for approximately 75% of cases of pregnancy-related thrombocytopenia. The platelet count drops by around 10% throughout the third trimester and returns to normal within six weeks after birth [6-8]. ITP was reported in 0.01-0.05 percent of pregnancies [9]. Moreover, it can be challenging in terms of diagnosis and management especially during pregnancy and can occur in all trimesters of pregnancy. Primary ITP is defined as thrombocytopenia without a known etiology or disease. Secondary ITP assumes the presence of an underlying illness causing immune dysfunction and thrombocytopenia. The majority of women with ITP have mild to moderate thrombocytopenia, and 30%-35% of cases necessitate intervention during pregnancy [5]. Pregnant women with thrombocytopenia are at risk of recurrence and the early diagnosis of the disease helps to reduce its complications. Against this backdrop, the following study was done to identify different causes of thrombocytopenia in pregnancy followed by its management in these patients, and to evaluate the outcomes in both, the mother and child, as no local data is specifically available from Pakistan during the COVID-19 era.

This article was previously presented as a meeting abstract at ISTH meeting, USA, on July 7-12, 2021.

## Materials and methods

Recruitment for this prospective, cross-sectional observational study of thrombocytopenia in pregnancy (platelet counts less than 100 x10^9^/L) was done from January 2017 to August 2020 at the National Institute of Blood Diseases (NIBD). The hospital's Institutional Review Board (IRB) approved this study, which was carried out in conformity with the Helsinki Declaration principles. Before participating in the study all patients gave written informed consent. To collect information about the patient's demographics and entire obstetrical history, a pre-designed structured performa was used. All patients were subjected to a clinical examination, routine laboratory testing, a complete blood count (CBC), and a blood film examination, liver function tests (LFTs), urea, creatinine, coagulation profile (PT, APTT), antinuclear antibodies (ANA), and viral serology including for hepatitis B, hepatitis C and human immunodeficiency virus (HIV) were performed. Similarly, dengue serology, malaria parasite (MP), and COVID-19 PCR were checked in patients with a history of fever along with C-reactive protein (CRP) and D-dimer. The stool for H. pylori antigen was also checked. Ultrasound of the abdomen was done to evaluate any visceromegaly, fatty liver, or other underlying pathology. For the study, thrombocytopenia was divided into three primary groups based on the severity of platelet counts at baseline: mild if the count was in the range 100-50x10^9^/L, moderate if the count was between 50-30x10^9^/L, and severe if the count was less than 30x10^9^/L. Women who had two platelet counts less than 100x10^9^/L during their pregnancy were eligible for the study From enrolment till delivery, the maternal platelet count was measured every 2-4 weeks; this was more frequent in patients of severe thrombocytopenia. Patients were excluded from the study if they had any of the following: missing platelet counts during pregnancy, birth, or post-partum; irregular follow-ups; hereditary causes of thrombocytopenia; or other causes including leukemia and bone marrow failure based on the findings from CBC, blood film and bone marrow examination or other workup done. The study's inclusion criteria for patients were pregnancy with thrombocytopenia such as GT was characterized as a healthy pregnant lady with no history of thrombocytopenia being diagnosed with thrombocytopenia for the first time during her pregnancy, had no other positive tests after assessments, and normalization of platelet count after delivery further confirmed the diagnosis. Those with a diagnosis of ITP or pregnancy with a previous history of ITP were defined as ITP in pregnancy [1]. Similarly, thrombocytopenia associated with hypertensive disorders (pre-eclampsia, eclampsia) is a major source of morbidity and mortality in pregnancy and was diagnosed using the Obstetrics and Gynecology criteria [2]. Moreover, other causes of thrombocytopenia such as acute fatty liver, infections such as malaria, hepatitis C, hepatitis B, HIV, H. pylori, COVID-19, thrombotic thrombocytopenic purpura (TTP) and hemolytic ureic syndrome (HUS) were included in the study. Patients with a platelet count of less than 30x10^9^/L or a bleeder's proclivity or had platelet count less than 50x10^9^/L after 36 weeks of gestation until delivery received treatment for all cases. Corticosteroid treatment included oral prednisolone (0.5-1mg/kg/day for 7-14 days) or intravenous (IV) methylprednisolone, dexamethasone (20mg/day for 5-7 days). When corticosteroid treatment was ineffective, intravenous immunoglobulin (IVIG) (400mg/kg/day for five days) was provided. During the pregnancy and perinatal period, patients who received corticosteroids, IVIG, azathioprine, or combination treatments and transfusion were included in the treatment group, while those who received blood transfusions, tranexamic acid, or iron supplements were included in the non-treatment group. The primary outcomes for this study were the maternal platelet counts at baseline/delivery and therapeutic response achieved by treatment regimen i.e. complete response at >100x10^9^/L, partial at 50-100x10^9^/L, and no response at below <50x10^9^/L platelet counts. Secondary outcomes of this study were composite on maternal antenatal/ postpartum bleeding, with hemoglobin and platelet counts monitoring whereas other secondary outcomes include; C-section, normal delivery, pre-term, abortion, neonatal thrombocytopenia, maternal death and fetal death were also assessed. Blood products were transfused before and after delivery. Neonatal platelet counts at nadir were also evaluated.

Statistical analysis

Statistical analysis was done by utilization of frequency, i.e., determined by percentile, was recorded for qualitative variables pertaining to the cause of thrombocytopenia and associated problems. The descriptive analysis such as, Mean ± Standard deviation (SD) was collected for quantitative variables such as gestation age, hemoglobin, total leukocyte counts (WBC), platelet counts. Chi-square was applied to estimate the outcomes of treatment and the non-treatment groups followed by the cause of thrombocytopenia. Similarly, paired t-test was applied to assess the association of mean platelets counts at diagnosis, before delivery and after delivery. A scatter plot was applied to assess the mean difference between neonatal platelet counts at delivery and with maternal platelet counts. SPSS version 23 (IBM SPSS, Inc., Chicago, IL, USA) was used to enter and analyze all of the data.

## Results

In the study period, a total of 350 cases of thrombocytopenia were reported at our institute of which 150 (42.8%) pregnant women were recruited based on inclusion and exclusion criteria. The patient's mean age was 27.3 + 4.64 years and the mean gestational follow-up was 29.91±7.94 weeks. Pallor 65.9%, bruising 23.25%, edema 9.3%, epistaxis 4.7%, and Malena 2.3% were the clinical symptoms at baseline. Mean hemoglobin was (mean ± SD) 10.5±3.7 g/dL, total leukocyte count was 13.2 ± 4.7x10^9^/L, and at baseline, the mean platelet count was 48.0±24. Categorically, platelet counts were observed at diagnosis as mild thrombocytopenia in 63 (42%) patients, moderate counts in 65 (43.3%), and severe < 30x10^9^/L in 22 (14.6%) patients.

Out of 150 confirmed cases of thrombocytopenia, causes related to pregnancy were identified in 95 (63.3%) patients. In this, GT was reported in 72 (48%) cases; fatty liver in five (3.3%) patients; pre-eclampsia in 11 (7.3%), and eclampsia in seven (4.6%) patients. Out of 72 cases of GT, supportive care treatment was given to manage thrombocytopenia in these patients which include IV tranexamic acid in 26 (36.1%) cases of mild bleeding while the rest were closely monitored and given supplements. Of the 18 women with preeclampsia or eclampsia, three (16.6%) patients received platelet transfusions before delivery due to antepartum bleeding; five (27.7%) due to platelet counts <50x10^9^/L before or during C-section; and 10 (55.5%) received transfusions after delivery due to postpartum bleeding.

Secondary causes of thrombocytopenia (not related to pregnancy) were diagnosed in 55 (36.3%) patients; idiopathic thrombocytopenic purpura (ITP) in 30 (20%); hepatitis C and nutritional insufficiency were both identified in nine (6%) of the patients; dengue in four (2.6%) and malaria in three (2%) patients; however, no case of hepatitis B, HIV, and COVID-19 was reported in our patients. Causes with maternal platelet counts at diagnosis are displayed in Table 1.

**Table 1:** Maternal platelet counts at diagnosis according to the causes of thrombocytopenia ITP: Immune Thrombocytopenic Purpura

Maternal platelets counts at presentation (n=150)				
Primary thrombocytopenia specific to pregnancy (n=95)				
CAUSES OF THROMBOCYTOPENIA	MILD 50-100x10^9^/L	MODERATE 50-30x10^9^/L	SEVERE <30x10^9^/L	TOTAL
	(n=63)	(n=65)	(n=22)	(n=150)
Gestational thrombocytopenia	40	32	0	72
Fatty liver	3	2	0	5
Pre-eclampsia	1	4	6	11
Eclampsia	1	2	4	7
Secondary thrombocytopenia not specific to pregnancy(n=55)				
ITP	7	13	10	30
Hepatitis C	4	4	1	9
Nutritional deficiency	5	3	1	9
Dengue	1	3	0	4
Malaria	1	2	0	3

Out of 30 ITP patients, 20 (66.6%) received treatment; oral prednisolone in eight (40%), IV methylprednisolone in three (15%), dexamethasone in two (10%); IVIG in two (10%) patient; while combination therapy of prednisolone with IVIG was given in five (25%) patients. Response to treatment of ITP patients is demonstrated in Table 2.

**Table 2:** Response to treatment in ITP patients IVIG: Intravenous immune globulin, IV: Intravenous

TREATMENT	COMPLETE RESPONSE	PARTIAL RESPONSE	NO. RESPONSE
Prednisolone (oral)	5(25%)	2(10%)	1(5%)
Methylprednisolone (IV)	2(10%)	1(5%)	0
Dexamethasone	2(10%)	0	0
IVIG	2(10%)	0	0
Prednisolone + IVIG	3(15%)	1(5%)	1(5%)

Among ITP patients, five (16.6%) received transfusions before delivery due to antepartum bleeding. Moreover, transfusions were done after delivery due to post-partum hemorrhage reported in six (20%) patients with a decrease in hemoglobin up to 7 g/dL. Similarly, out of seven (23.3%) patients of mild thrombocytopenia; two (6.6%) cases had a decline in the platelet counts and were treated with prednisolone and showed complete response to treatment. Ten (33.3%) patients received transfusions due to low platelet counts <30x10^9^/L at the time of delivery due to C-section. Neonatal thrombocytopenia was reported in three (10%) newborns (range 60-73x10^9^/L) among these ITP patients.

Other remaining patients in this group were treated with supportive care including treatment of infection (anti-malarial), supplements (iron, vitamin-B12, and folic acid) and their platelet counts were closely followed. Out of nine (6%) cases, one (11.1%) patient of hepatitis C with platelet count <30x10^9^/L received transfusion at the time of delivery. Overall, neonatal mean platelet counts 197.26±44.7 at nadir were significantly (P=<0.000) different with maternal mean platelet counts 70.30±65.4 at delivery. Out of the total of 150, 78 (52%) were through normal delivery while 67 (44.6%) patients underwent C-section. Also, five (3.3%) abortions took place but no obvious cause was identified. Maternal outcomes are shown in Table 3.

**Table 3:** Maternal and neonatal complications and outcomes in patients of thrombocytopenia (n=150) C-Section: Caesarean section

OUTCOMES	SPECIFIC TO PREGNANCY	NOT SPECIFIC TO PREGNANCY
MATERNAL COMPLICATIONS		
Antepartum bleeding	6(4%)	8(5.33%)
Postpartum bleeding	15(10%)	6(4%)
Maternal death	0	0
MATERNAL OUTCOMES		
Normal delivery	61(40.6%)	17(11.33%)
C-Section	52(34.6%)	15(10%)
Abortion	3 (2%)	2(1.3%)
NEONATAL OUTCOMES		
Neonatal thrombocytopenia	0	3(2%)
Pre term	4(2.6%)	1(0.66%)
Fetal death	0	0

Overall, most common side effects reported were hypertension in 14 (9.3%), hyperglycemia in 11 (7.3%), weight gain in 10 (6.6%), tachycardia in six (4%), myalgia in five (3.3%), peptic ulcer in four (2.6%), osteoporosis four (2.6%), and hypotension in two (1.33%) patients.

## Discussion

In our study, the most common cause of thrombocytopenia (48%) was GT, which had a favorable pregnancy outcome even in the third trimester; this was followed by ITP (20%) and hypertensive disorders in pregnancy (12%). Based on clinical experience, GT has so far proven to be safe. However, the precise mechanism of its etiology remains unknown. Most researchers believe that GT is associated with hemodilution or rapid platelet clearance [9,10]; in this situation, there is no destruction of platelets but a relative fall in platelet numbers. As a result, the clinical feature is primarily moderate thrombocytopenia, which usually manifests itself in the second or third trimester of pregnancy. In our cohort of 150 women, 72 GT patients had a platelet count of more than 30x10^9^/L, and thrombocytopenia was first diagnosed in virtually all of these patients during the second or third trimester of pregnancy; our findings are consistent with those of other studies [9,11].

Most obstetric patients with ITP have a history of previous thrombocytopenia, and the majority of cases are discovered during the first trimester of pregnancy [12,13]. ITP is an autoimmune disease, affecting platelet survival, but also platelet production. A previous transient episode of thrombocytopenia, underlying autoimmune illness, or severe thrombocytopenia increases the risk of an ITP diagnosis [14]. Similarly, all our 30 patients had a prior history of ITP with 23 patients having moderate to severe thrombocytopenia at baseline and presenting in the first trimester of pregnancy. They were on Azathioprine or Eltrombopag (thrombopoietin receptor agonists) or combination therapy. Due to pregnancy, Eltrombopag and Azathioprine were stopped and these 23 patients (76.6%) received treatment with different steroids and combination therapy with complete response in 46.6% patients; partial response in 13.3%. This shows that overall these patients had a good response to steroids while only two patients in our study received IVIG because of the high cost of this medicine. All the remaining patients were closely followed and counseled for their thrombocytopenia and received supplements. Moreover, ITP can occur at any time during the pregnancy, with varying degrees of thrombocytopenia, and platelet count may drop further as the pregnancy proceeds, improving postpartum. The risk of bleeding is higher with platelet counts less than 20x10^9^/L [2-5]. Similarly, our study demonstrated that 10 patients had severe thrombocytopenia (<30x10^9^/L), among them six patients had antepartum bleeding, whereas four had postpartum bleeding which was managed with I.V tranexamic acid and platelet transfusions. Three (2%) newborns had neonatal thrombocytopenia but with no evidence of bleeding. This is consistent with earlier studies that have found that thrombocytopenia in newborns is uncommon (about 10% to 15%) and does not result in bleeding issues [15-17]. Thus, the outcome of pregnancies with thrombocytopenia in our study was overall favorable and comparable with zero mortality and this was mainly accomplished with the help of a multidisciplinary team including hematologists, obstetricians, and a pediatrician at our center. Similarly, in the treatment group, no severe newborn abnormalities were found.

Thrombocytopenia due to pregnancy is associated with hypertensive disorders such as preeclampsia and eclampsia; these were the third common cause (12%) in our study. Activation of both the coagulation and fibrinolytic systems causes severe thrombocytopenia and disseminated intravascular coagulation (DIC), which happens in some preeclampsia patients, and also plays a role in encouraging platelet activation and rapid clearance [18]. We observed that in our 70 multigravida patients it was associated with severe thrombocytopenia with preterm deliveries in five patients. They all had postpartum bleeding which was timely managed with pack red cells and platelet transfusions and supportive care measures. Fortunately, no fetal/maternal morbidity and mortality were reported in our study. However, complications associated with this disorder are reported by other workers [4,19-21].

Other minor causes of thrombocytopenia were infections such as hepatitis C, dengue, and malaria. Platelet activation, splenic pooling, and a reduced platelet life-span of 2-3 days (versus typical 7-10 days) all contribute to lower platelet counts during infections [22,23]. The significance of immunological factors is unclear because the fall in platelet count is directly proportionate to disease severity and rebounds quickly with infection recovery [24]. Only one woman with hepatitis C in our study had severe thrombocytopenia with spontaneous bleeding at the time of delivery, which was treated with platelet transfusion.

In Pakistan, the first COVID-19 case was reported in February 2020. Since then, the pandemic has continued to wreak havoc on the country's already overburdened healthcare system [25]. Many studies suggest that clinical manifestation of COVID-19 in pregnancy is not different from non-pregnant adults, yet, according to a few studies pregnancy can complicate the clinical course of COVID-19 [26]. Since our study does not report any COVID-19 case, therefore, no such complication was observed in these patients.

Furthermore, there were 44.6% (67/150) of women who had a cesarean section, which was determined according to the maternal platelet, fetal intrauterine condition, maternal complications, and other obstetric indications. Platelet counts more than 50x 10^9^/L provide for adequate hemostasis during spontaneous vaginal delivery and cesarean section [27]. With platelet count within 20-50×10^9^/L, vaginal delivery was considered underestimated that the labor was controlled within 12 hours with adequate blood components and close monitoring.

The therapeutic choices for thrombocytopenia in pregnancy are limited and primarily established by clinical experience [2]. The mean difference in platelet counts in our study at baseline and after delivery showed significant difference P<0.001. Platelet count was higher after birth than during pregnancy. Although patients were followed every 2-4 weeks in the study from enrolment to delivery, their platelet counts were reported at baseline and delivery due to their enrolment at different time points during the gestational period. Thus, careful and regular assessments of clinical features and management of platelet counts are critical in these patients to reduce the risk of bleeding for both mother and child.

The limitation of our study is that it is a single-center; however, this sort of large local prospective study is the first in our knowledge to analyze the many causes of thrombocytopenia in pregnancy and their treatment during the COVID-19 period. To maintain objectivity of the study, the participants were recruited from a referral care hospital with a vast catchment area distributed across various urban and semi-urban regions of Sindh province; thus, the sample was likely representative of a large segment of the Pakistani population. Moreover, we need more local studies to better characterize our patients, with proper monitoring of blood counts and their treatment to fully understand the impact of COVID-19 on this group.

## Conclusions

To summarize, our findings reveal that preeclampsia and eclampsia are dangerous illnesses with a substantial risk of consequences. GT is benign and is the most common cause of thrombocytopenia in our study and does not need active treatment at all, whereas the other causes such as ITP, infections, and nutritional deficiency all demand individualized management. Hence, timely identification of causes and management is important both for the mother and child to prevent complications.
